# Relationship Between the Type of Allergen and Night Sweat in Allergic Children

**DOI:** 10.7759/cureus.48174

**Published:** 2023-11-02

**Authors:** Ayşe Kırmızıtaş Aydoğdu, Ali Özdemir

**Affiliations:** 1 Pediatric Allergy and Immunology, Mersin City Training and Research Hospital, Mersin, TUR; 2 Pediatric Pulmonology, Mersin City Training and Research Hospital, Mersin, TUR

**Keywords:** allergic rhinitis, asthma, sweating, allergen, house dust mite

## Abstract

Background: Atopic diseases can accompany allergic sensitization. The amount of allergen is as important to know since allergen exposure affects sensitization development. Some allergic children complain of intense sweating during the first few hours of transition to sleep especially at night. This study was carried out to investigate the relationship between the type of allergen to which children are sensitized and night sweating.

Methods: Children aged two to 18 years old with single allergic sensitization were included. A specific immunoglobulin E (IgE) skin prick test results were obtained from the medical records of the patients. Then, the patients’ families were asked to evaluate the levels of sweating from 0 to 10 with the Visual Analogue Scale (VAS) questionnaire and the Sleep Disturbance Scale for Children (SDSC).

Results: Sensitization to mites was more prevalent in the group of patients with night sweating (p<0.001), and pollen allergy was more prevalent in the group of patients without night sweating (p<0.001).

Conclusion: The amount of mites and wetness in bed sheets might be responsible for allergic sensitization. A change in clothes and bed sheets for those with intense sweating might ensure better sleep and reduce the severity of the atopic symptoms. Such a recommendation might provide a better clinical outcome in these patients.

## Introduction

Atopic diseases, including asthma, allergic rhinitis, atopic dermatitis, and urticaria, are generally accompanied with allergic sensitization. Sensitivity to an inhaled allergen, such as pollen, house dust mites, animal epithelium, or mold, is commonly observed in this group of patients. The amount of allergen is as important to know since exposure to the allergen affects allergic sensitization development. The more a person is exposed to an allergen, the more likely he or she develops sensitization. Current evidence provides consistent support for a threshold level of exposure of 2 µg of group 1 mite allergen (or about 100 mites/g of dust); above this level, the individual becomes highly at risk of developing allergic sensitization. Furthermore, a dose-response relationship exists between the mean concentration of mite allergen in houses, and this sensitization is important for the risk of asthma [1-3]. Therefore, preventing or reducing the contact of patients with allergens is an important step, in addition to drug therapy, to control the symptoms of the disease. Thus, most clinicians recommend an exposure reduction plan to indoor allergens to their atopic patients [4-9].

The main sources of mites indoors are woolen products, such as bedclothes, sheets, pillowcases, blankets and carpets, fluffy toys, zigzag parts of fabric-covered furniture, and curtains. Mites obtain nutrients that are necessary for them to survive from human skin and human hair. Since they are unable to search or drink water, they need the humidity in the environment to meet their water needs [6]. The amount of house dust mites is lower in dry climates and high altitudes, while it is higher in coastal areas and highly humid areas. Although it is a common practice to measure ambient humidity, it is the humidity within carpets, sofas, mattresses, or clothing that is relevant [1]. Mite allergens do not float in the air, but they are often found in dust on the ground. The amount of allergen per gram of settled dust, not in air samples, gives more accurate results about mite density [6]. 

Some allergic children complain of intense sweating during the first few transitional hours of sleep, especially at night. Night sweating (sleep hyperhidrosis) is a general episode of hyperhidrosis that occur during sleep, and its severity can change from mild to heavy sweating that may require change in beddings [10,11].

Allergen sensitization-related night sweating is unclear in atopic children. Therefore, we aimed to investigate the relationship between the type of allergen sensitization and night sweating in patients with atopic dermatitis, urticaria, and asthma.

## Materials and methods

One-hundred eleven patients with doctor-diagnosed atopic diseases who were followed at the Pediatric Allergy Section of Mersin City Training and Research Hospital, Mersin, Turkey, with a duration of two years were included and retrospectively evaluated in the current study. The time of diagnosis, age, gender, eosinophil count, total IgE, food mix specific IgE (fx5), phadiatop (inhalant allergen mix specific IgE), and skin prick test were obtained from the patients' records. This group was compared with non-atopic and healthy children in terms of night sweating. Forty-nine healthy children were evaluated as a control group, and their sweating characteristics were evaluated. Then, the families of children with a diagnosis of atopic dermatitis, urticaria, and asthma were invited to a Visual Analogue Scale (VAS) and questionnaire that was designed for night sweating scoring [12] and healthy children. This score consisted of 0 to 10 points related to the intense of night sweating. At the same time, night sweats were recorded using the items related to night sweats in the Sleep Disturbance Scale for Children (SDSC) questionnaire by asking them to rate night sweats on a five-point scale (1-5). Night sweating was evaluated using the items related to night sweats (include falling asleep sweating and night sweating) with the survey. Subjective report of nocturnal sweating on a frequency scale of 1-5: (1) never, (2) occasionally (once or twice per month or less ), (3) sometimes (once of twice per week), (4) often (3 or 5 times per week), and (5) always (daily) [13].

Skin prick test consisted of allergens associated with *Dermatophagoides pteronyssinus* (DP), *Dermatophagoides farina* (DF), meadow and grain pollen mix, weed pollen mix, tree pollen mix, olea pollen, alternaria, cockroach, cat and dog epithelium, milk, egg, wheat, soy, peanut, hazelnut, beef, chicken, and fish mix antigens. The test was applied to the forearm volar aspect.

Histamine hydrochloride (1 mg/ml) was used as a positive control, and a physiological saline solution was used as a negative control. The result was evaluated after 15 minutes. For histamine reaction, edema of greater than 5 mm accompanied by erythema was stipulated. A mean induration diameter of ≥3 mm from the negative control in the skin test and concomitant erythema was considered positive [14].

Serum-specific IgE levels of the patients were studied by the ImmunoCAP method (Phadia, Uppsala, Sweden). A value of ≥0.35 kU/l was considered positive for food- and/or inhalant-specific IgE. Specific IgE levels of food allergens (i.e., egg, milk, fish, wheat, peanut, and soy bean) were evaluated with Fx5, and aeroallergen serum-specific IgE levels were evaluated with Phadiatop. Eosinophilia was defined when the eosinophil level was above 4% in the complete blood count.

After an informed consent was obtained from the caregivers of children that qualified for the study, they were questioned to evaluate their children’s sweating levels by the SDSC and VAS questionnaire [12,13]. This score consisted of questions related to the characteristic sweating, whether mild or excessive, day or night, whole or partial of the body, and at beginning or all night in their child. A heavy of existence of hyperhidrosis was scored “10” and “0” if none according to VAS questionnaire. Night sweats were assessed using the SDSC by asking them to rate on a five-point scale (1-5) [12]. The study protocol was approved by the Toros University Clinical Research Ethics Committee (approval no. 49). 

Statistical analysis

IBM SPSS Statistics for Windows, version 23 (released 2015; IBM Corp., Armonk, New York, United States) was used in the statistical analysis of the data. Categorical measurements were summarized as numbers and percentages. Continuous measurements were summarized as mean and standard deviation (median and minimum-maximum, when needed).

During the comparison of categorical expressions, chi-square and Fisher exact tests were used. Shapiro-Wilk test was used in determining whether the parameters are normally or not normally distributed. Independent student t-test was used for parameters that showed a normal distribution. A P-value of less than 0.05 was considered statistically significant.

## Results

Only mite allergy was observed more frequently in the patients with night sweats (p<0.001), and pollen allergy was observed less frequently in the patients with night sweat than the patients without night sweats (p<0.001; p<0.05). No significant difference was found between other allergy types among the compared groups. In addition, there was no significant difference between the groups with/without night sweats and the age, gender, asthma, allergic rhinitis, urticaria, and atopic dermatitis variables of the patients (p>0.05) (Table 1).

**Table 1:** Differences between the introductory findings and groups

	Patients without night sweat (n=72)	Patients with night sweat (n=39)	Total number of patients (n=111)	Controls (n=49)	p^c^
	n(%)	n(%)	n(%)	n(%)	
Age	8.57± 3.88	8.25±3.64	8.52±3.85	8.40±3.76	0.990
Male	41 (56.9)	26 (66.7)	67 (60.4)	29(%59.1)	0.317
Female	31 (43.1)	13 (33.3)	44 (39.6)	20(%40.9)	
Asthma	30 (41.7)	18 (46.2)	48 (43.2)		0.649
Allergic rhinitis	43 (59.7)	29 (74.4)	72 (64.9)		0.123
Urticaria	21 (29.2)	6 (15.4)	27 (24.3)		0.106
Atopic dermatitis	12 (16.7)	4 (10.3)	16 (14.4)		0.359
Mites	17 (23.6)	33 (84.6)	50 (45.0)		<0,001**
Pollen allergy	45 (62.5)	2 (5.1)	47 (42.3)		<0,001**
Mold	10 (13.9)	2 (5.1)	12 (10.8)		0.156
Pets	3 (4.2)	-	3 (2.7)		0.196

The prevalence of Phadiatop (p=0.010) and total IgE >1 in patients with night sweats (p=0.048) was found to be significantly higher than in patients without night sweats (p<0.05). However, there was no significant difference between the other parameters and the groups with/without night sweats (p>0.05). While the VAS values were reported between 8 and 10 in the group of patients with positive night sweat, they were reported between 0 and 2 in the group of patients with negative night sweat (Table 2). 

**Table 2:** Differences between allergic findings and the groups Fx5: food mix specific IgE

	Patients without night sweat (n=72)	Patients with night sweat (n=39)	Total number of patients (n=111)	p^c^
	n(%)	n(%)	n(%)	
Fx5				
Negative	19 (67.9)	11 (78.6)	30 (71.4)	0.469
Positive	9 (32.1)	3 (21.4)	12 (28.6)	
Phadiatop				
Negative	17 (53.1)	2 (13.3)	19 (40.4)	0.010*
Positive	15 (46.9)	13 (86.7)	28 (59.6)	
Total IgE				
<100	14 (35.0)	3 (12.5)	17 (26.6)	0.048*
>100	26 (65.0)	21 (87.5)	47 (73.4)	
Eosinophil				
	29 (51.8)	15 (55.6)	44 (53.0)	0.747
>%4	27 (48.2)	12 (44.4)	39 (47.0)	
Food prick test	8 (11.1)	3 (7.7)	11 (9.9)	0.565
Fish	1 (12.5)	-	1 (9.1)	0.666
Hazelnut	1 (12.5)	-	1 (9.1)	
Milk	2 (25.0)	-	2 (18.2)	
Egg	4 (50.0)	3 (100)	7 (63.6)	

## Discussion

Inhalant allergen sensitivity is common in atopic diseases, and the severity of the diseases might enhance in the presence of heavy exposure to inhaled allergens. Preventing or reducing the contact of patients with the allergen is an important step in controlling the symptoms of the diseases and the drug therapy [4-9]. In the current study, we observed that there was a correlation between the type of allergen sensitivity and night sweating in atopic children. In addition, children with mite allergy sensitivity were more prone to suffer night sweating than other allergens. To our knowledge, such a relationship has not been investigated in the literature.

There are a limited number of studies on the incidence of night sweats [14-16]. In a study investigating the prevalence and characteristics of frequent night sweats in obstructive sleep apnea patients, the prevalence of frequent night sweats in the general population was found to be 9.6% in men and 12.8% in women [17]. In a study conducted with children, 11.7% of children were found to have night sweats. It was found that allergic diseases, such as asthma and allergic rhinitis, were more common in children with night sweats. However, there is no information on the allergen sensitivity detected in these patients [18].

A study conducted by Oflu et al. [19] revealed that sweating increased in children with allergen sensitivity. However, the type of allergens was not compared in detail in this study. It is well known that mites are creatures that do not have moisture metabolism and depend on external humidity in order to survive. A major problem with any assessment of exposure to dust mite antigens is that patients often sleep or sit with their heads very close to mite-infested material (e.g., sofas, blankets, pillows, and carpet). The use of personal monitors close to the breathing zone has been routinely used to gather accurate information [1,20,21]. In this respect, Vackova et al. [22] carried out a study regarding the room temperature whereby mites prefer living. They reported that mites preferred living at a temperature of around 32-36 °C. This finding might explain why house dust mites on mattresses preferred to live close to humans. 

Our study did not include patients with multiple sensitivity, as if it might make for a more difficult comparison. When the patients compared for single sensitivity to mite, pollen, alternaria, and animal epithelium, night sweating in children with mite sensitivity was more prominent than others. The parents reported that their children sweated intensely enough to leave the bed soaked in the first two hours of asleep. However, they also reported that their children remained dry afterward until the morning. Based on our study results, children with mite allergy might facilitate the development of sensitization as the amount of mites in the bed could increase with humidity. As it was expressed by the families in our study, patients with mite allergy did not sweat all night long and generally only sweated within the first two hours of falling asleep. Following this intense sweating process, it could be recommended to change both the patients’ clothes and the bed sheets in order to reduce the amount of mite allergen exposure. Other measures, such as the use of acaricide sprays, might also help prevent mites from growing on bed sheets with this respect. Such an approach might alter the course of allergic sensitization in atopic children at an early stage [23,24]. As a result, the need for both a clinical visit and allergy medication may help reduce allergen response. Further research to approve these assumptions is needed to determine whether these methods would help alleviate symptoms. It should be noted that the main limitation of our study was single-centered in origin with a limited number of patients and sweating was just evaluated by self-reporting from families of atopic children.

## Conclusions

Children with mite sensitivity are prone to night sweating particularly in the first two hours of asleep. It could be said that reducing exposure to mite allergen in atopic diseases might be important in terms of reducing the severity of the diseases and may be beneficial to prevent unnecessary drug use and clinic visits. Further studies with larger numbers of patient groups are needed to confirm our findings.
